# An Integral Pedagogical Strategy for Teaching and Learning IoT Cybersecurity

**DOI:** 10.3390/s20143970

**Published:** 2020-07-17

**Authors:** Julia Sánchez, Adrià Mallorquí, Alan Briones, Agustín Zaballos, Guiomar Corral

**Affiliations:** Engineering Department, Universitat Ramon Llull (URL), La Salle, 08022 Barcelona, Spain; adria.mallorqui@salle.url.edu (A.M.); alan.briones@salle.url.edu (A.B.); agustin.zaballos@salle.url.edu (A.Z.); guiomar.corral@salle.url.edu (G.C.)

**Keywords:** IoT, cybersecurity, learning methodology

## Abstract

Internet of Things (IoT) has become a fundamental content of any engineering program due to the emerging need of experts in this field. However, the complexity of technologies that interact in IoT environments and the amount of different professional profiles required to design, implement and manage IoT environments, considering cybersecurity as a must, has led to a huge challenge in the educational world. This paper proposes an integral pedagogical strategy for learning IoT cybersecurity structured in three different stages, in a higher education institution. These stages focus not only on the content about IoT and cybersecurity but also on the competencies to acquire, the most suitable learning methodologies and the expected learning outcomes. The association of these concepts in each stage is detailed. Examples of courses are explained, the related competencies and learning outcomes are specified, and the contents and methodologies to achieve the expected results are described. An analysis of student results and stakeholder evaluations is provided to verify if the pedagogical strategy proposed is suitable. Furthermore, students’ feedback is included to corroborate the innovation, the suitability of the acquired skills, and the overall student satisfaction with the related courses and consequently with the proposed IoT cybersecurity pedagogical strategy.

## 1. Introduction

The IoT (Internet of Things) paradigm has gained importance in recent years, mainly because of the benefits it provides, as it can improve our quality of life by helping us make difficult decisions more efficiently, or provide intelligence to the devices around us to perform daily tasks with minimal human intervention. IoT turns homes, buildings, hospitals, cities, and industries, among others, into intelligent systems capable of obtaining knowledge of the environment, and applying it for adaptation according to the needs of the inhabitants.

In order to obtain these benefits, IoT interconnects people, objects, and devices (vehicles, appliances, sensors, actuators, work equipment, laptops, smartphones, PDAs, etc.) so that they can communicate smartly with each other, or with people. IoT devices acquire data from the environment, understand what is happening, and respond according to current needs. Therefore, it is necessary to process the data which can be carried out on the devices themselves or more commonly in spaces such as the Cloud, where computing power is higher (necessary if there is a lot of information to process in a short time). As a result of processing this data, devices are able to make autonomous decisions or provide information to people so that they can make better decisions about their daily activities (whether in a work or personal environment).

Interconnected ‘Things’ can collect very sensitive information from people or work environments and, therefore, the privacy of data and the security of all their traffic on the Internet has become a major concern. For this reason, cybersecurity in the IoT environment has become increasingly important in recent times.

IoT has come to stay, and given the importance it currently has, along with the complexity of the environment needed to obtain the aforementioned features, learning how to develop secure IoT systems has become a significant challenge. There is a high demand for professionals with both IoT and cybersecurity skills in today’s world.

In order to prepare university students with these new technologies and their use, education needs to be able to incorporate effective learning techniques.

Teaching these two topics is a challenge today, given the variety of existing techniques that can be applied.

Various techniques and approaches have been used to transfer the necessary IoT and cybersecurity skills to university students. In [1], a model is proposed for conducting IoT classes based on a web-service oriented cloud platform. The pilot provides students with knowledge about IoT concepts, possibilities, and business models, and allows them to develop basic system prototypes using general-purpose microdevices and a cloud and service infrastructure. This approach reflects the vast current trend of class virtualization and access to content at any time and from anywhere that other areas of study have also taken place. In [2], an approach is proposed as well as the framework for teaching wireless sensor networks and related technologies with the Arduino platform. This framework explains and reviews the critical communication elements of IoT architecture and communication networks, but it has also been shown that there may be some reluctance to teach IoT through practice. As mentioned in [3], “the main goal of university-level education is to teach long-lived principles and concepts, and not short-lived systems or tools”. Here, researchers show another perspective on the same problem, but focus on providing students with needed knowledge, concepts, and principles of IoT, without using one particular hardware or software. They think that “since the students learn many different platforms and abstract away from their details, they might not become aware of all concepts and properties of the Internet of Things”. So, they identify a list of the most important concepts and properties and how they could integrate them into the syllabus. They also incorporate some hands-on labs to support essential concepts.

Regarding cybersecurity, different approaches for teaching and learning exist. Authors in [4] present an insight into current approaches taken in the practical education of cybersecurity, and give requirements and best practices for future training platforms based on the defined teaching process. This teaching process presents real-world problems to students, to demonstrate their technical skills, to solve problems implemented into physical laboratories, simulation laboratories (video game, scenario-based), or virtual laboratories (desktop-based, Cloud-based with a single virtual machine (VM) or multiples VMs). In this case, the relevant contribution of researchers is to establish the requirements of the practical exercises to be profitable.

The participation and motivation of students in their learning process are some of the great challenges to be achieved by instructors. Thus, gaming experiences are gaining ground for the teaching of cybersecurity. Researchers in [5] present an experiment to measure the validity of an immersive gaming experience derived from Escape the Room (ETR) challenges of similar composition. A week-long experience is prepared with different activities to perform, where participants locked in a physical location must solve different probes to escape, based on cybersecurity concepts. Researchers state that these methods are effective for cybersecurity education. Other researches prove that applying initiatives such as CTF (Capture The Flag) competitions encourages students to achieve the desired learning objectives, as stated in [6]. 

Acquiring cybersecurity competencies is not only attractive in higher education. Secondary education institutions are also beginning to incorporate initiatives so that younger students begin to gain knowledge on this topic. Authors in [7,8] present good results when using a networked robotics environment to accompany cybersecurity learning, such as RoboScape [8], using NetsBlox as a visual programming environment to introduce students to distributed computation and computer networking. 

There are multiple alternatives to training university students in IoT and cybersecurity. Still, most of them detail specific methodologies to address specific concepts and objectives during a subject or a short period of time. At La Salle (Ramon Llull University) [9], we conceive IoT cybersecurity learning on all stages of the learning process and through a comprehensive strategy that addresses both specific competencies for IoT and cybersecurity and generic competencies or soft skills. We consider that these soft skills (like making decisions, commitment, interpersonal communication, flexibility, time management, leadership, creativity and problem solving, teamwork, responsibility, and capability to work under pressure) are also essential to obtain the valuable engineer profile that the market demands. 

This paper presents an integral pedagogical strategy for teaching and learning IoT cybersecurity for network architectures in a higher education institution. In Section 2, the pedagogical strategy, based on the acquisition of competencies, is presented. Moreover, the specific and generic competencies (technical and soft skills) are defined, and the learning methodologies and outcomes are exposed, distributing all of them between stages of the learning process. In Section 3, the methodology of the two subjects employed to acquire IoT cybersecurity knowledge in early stages is explained, and some examples of tasks performed are provided. Additionally, competencies and learning outcomes are redistributed between the two subjects, to clearly expose which ones are related to each subject. In Section 4, activities performed in medium stages are explained, and competencies and learning outcomes are divided between the two main activities, collaboration in research groups and undergraduate program final thesis elaboration. In Section 5, the Master as a Service (MaaS) initiative is presented as the advanced stage activity performed to acquire new competencies, mostly soft skills that help to shape valuable engineers. In Section 6, the results and feedback of students in each stage are discussed. Finally, in Section 7, conclusions of the work developed are drawn.

## 2. Competency-Based Learning for IoT Cybersecurity

### 2.1. Evaluation of Academic Competencies

Within the European Higher Education Area (EHEA), all undergraduate or postgraduate university programs must have, in their study plans, a set of competencies that must be practiced and evaluated. In fact, the competency model should be the pillar where all the degrees develop their teaching-learning programs [10,11,12]. If we analyze the competency model, we find two types of competencies: generic and specific ones. Generic competencies are the capacities that can be acquired in any kind of study, while the specific ones are those of the scope of knowledge of the program.

The evaluation by competencies has gained more traction in teaching as an important element to be able to have a more in-depth knowledge of the student and their aptitudes and abilities. The competencies have been implemented as an evaluation method both in primary and secondary education, as well as in university studies. Quality agencies, those performing the evaluation, accreditation, and certification of quality in the field of universities and higher education institutions, have published a lot of documentation, with guidelines on how to apply a competency-based assessment. For example, in Spain, there are guides for evaluation by competencies in areas as diverse as Technology and Architecture [13], Social Education [14], Humanities [15], the Science of Physical Activity and Sports, the Social Sciences [16], or Medicine [17] to cite some of the relevant ones. A competency-based teaching model is presented in [18].

Although much work has been done in the development of competencies within the curricula of undergraduate and postgraduate university programs, their evaluation continues to be an unsolved aspect. Probably, there are different causes that lead to this situation. Perhaps the main one is that the emergence of competencies and learning objectives clashes head-on with the heritage of curricula that are content-centered. Additionally, most undergraduate and postgraduate programs focus on the development and evaluation of the specific competencies of their degree. If the focus is on postgraduate programs, where most of these have a professionalization character and are oriented to good job placement, we should ask ourselves if these specific competencies defined in each of them are those that the market demands.

#### 2.1.1. Types of Generic Competencies

As stated before, Project Tuning [19], since it is one of the most widespread in the university field, it classifies the competencies into two broad groups: generic and specific. The generic competencies are divided into three types: instrumental, interpersonal, and systemic. The most outstanding capacities for each type of generic competency are the following:

● Generic instrumental competencies; this type of competencies includes cognitive, methodological, technological, and linguistic abilities.
● Generic interpersonal competencies; these competencies describe social skills related to personal skills and ethical commitment.
● Generic systemic competencies; this type of competencies combine the understanding, the awareness, and the knowledge that allow students to see how the different parts of a whole are related and grouped.

One of the main challenges that exist in university studies is the need to transmit and evaluate these competencies accurately. Many of the technical degrees are focused on specific contents closely linked to the curricula, while leaving aside this type of more transversal skills. However, many of the jobs offered in the market require people trained in not only the most technical aspects but also employees with transversal skills that help the successful development of the different projects carried out in an industry. It is going to be analyzed in the following section.

#### 2.1.2. Transversal Competencies

Once the different typologies of competencies are explained, some references are presented to see what is demanded from the industrial sector and what future perspectives exist in this sense. If the competencies that graduate degrees could offer are to be contrasted with the so-called transversal skills most valued by employers, also known as soft skills, we find many reference reports that can indicate future trends in the sector.

The National Career Service, the government agency of the United Kingdom, released a report that cited the transversal skills most valued by employers. Reports like “Trabajar en 2033” [20], or the Adecco Report on the future of work in Spain 2016 [21] allow us to make a synthesis of the skills demanded from the business world.

Transversal skills are related to making decisions, commitment, interpersonal communication, flexibility, time management, leadership, creativity and problem solving, teamwork, responsibility, and capability to work under pressure. In other words, the ability to make decisions in an agile, informed and consistent manner is an essential skill for any job. Highly committed profiles do not usually require a lot of supervision to give the best and fulfill their tasks with reliability. Likewise, communication skills, oral and written, are highly valued qualities.

At the same time, knowing how to adapt to the circumstances and not fear new challenges is evidence of a flexible nature, capable of leaving one’s comfort zone and facing difficulties with a positive attitude. In this sense, the capability to know how to prioritize the most important tasks and delegate those that are not so important and the ability to understand how to respect deadlines, deal with crises and face the changes that may arise at the last moment are also well-valued by the employers.

Regardless of the job position, it is also positively valued to know how to motivate colleagues to give the best of themselves, how to work in a team, in an open, transparent and constructive way, and knowing how to assume one’s own mistakes, as well as successes. Finally, related to creativity and problem solving, the ability to address problems by trying to find solutions in a logical and creative way is an important factor in the recruitment stages.

If we cross the demands that come from the labor sector with the competencies described in the academic model, it is observed that there is a huge correlation between the two. So, why do we not face programs that focus on the capacities that the industry demands beyond the technical or specific capabilities that are considered to be included in the degree itself?

Universities are facing significant challenges. On the one hand, students should not stop acquiring the specific competencies related to their field of knowledge, but on the other hand, it is necessary that other qualities, many of them represented in transversal competencies, can be developed in class.

### 2.2. An Integral Pedagogical Strategy for Teaching and Learning IoT Cybersecurity

As explained in the previous section, university education grapples with the challenge of preparing professionals for a technological world in more competitive and globalized work environments. This new paradigm will require cross-disciplinary skills, such as critical thinking, problem-solving, resilience, and agile work in teams formed in a heterogeneous way. University must prepare our students for this looming scenario, for example, by strengthening the connection between what students learn and what the outside world is going to require from them (paraphrasing our founder, St. Jean-Baptiste de La Salle, “You cannot educate them better than giving them a good example”).

To achieve this goal of teaching IoT cybersecurity and assure that our students acquire the needed competencies for this globalized world, a complete pedagogical strategy that involves several learning methodologies is defined. These methodologies are applied through several subjects and activities performed inside the curricula programs. 

**Figure 1:**
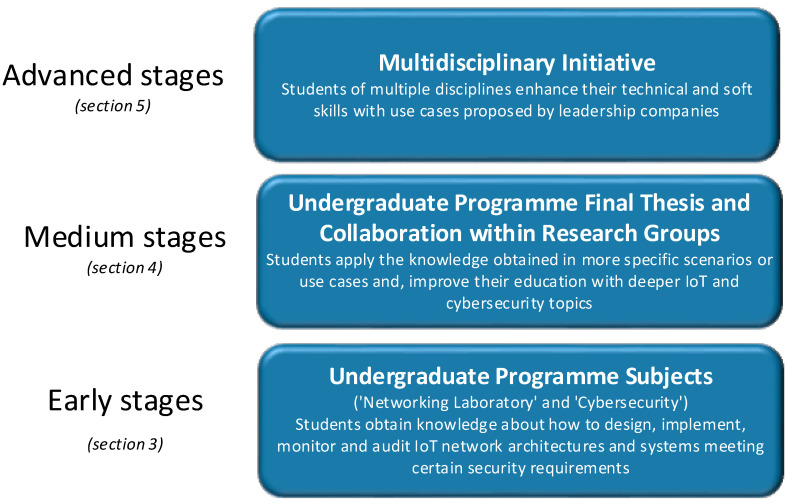
Pedagogical strategy for teaching Internet of Things (IoT) cybersecurity.

As we can see in Figure 1, the pedagogical strategy includes a continuous and step-by-step learning path, starting in the early stages, where undergraduate program subjects provide students with fundamental and technical IoT and cybersecurity knowledge, continuing in medium stages, where students collaborate with research groups for their final thesis elaboration or their interest in a deeper learning, and finishing in the advanced stages, where they take part in initiatives, sharing work activities with university mates of several disciplines.

In our university, we have a wide range of undergraduate programs in the field of engineering, in total seven different specialties (Computer, Telematics, Telecommunication Systems, Electronic Systems and Robotics, ICT Management, Audiovisual and Multimedia and Videogames). All curricula are designed so that students acquire both general competencies desirable for a valued engineer in the global market and specific competencies associated with their field of knowledge. Among all the programs, three stand out, to which the competencies for IoT and cybersecurity are directly associated; Computer Engineering, Network (Telematics) Engineering and ICT Management Engineering. Moreover, being somewhat more precise, Network Engineering students are those who acquire more competencies for IoT and cybersecurity learning since it is the program that prepares students to work specifically in the Network and Internet Technologies sector. In the early stages, apart from achieving technical competencies, the strategy proposes the assimilation of transversal competencies through joint work, in groups, between students from these three different specialties.

Moreover, at our university, different research groups are dealing with several technological fields; internet technologies, data science, storage and big data, technologies applied to learning, and multimedia technologies, among others. Within the proposed pedagogical strategy, in the medium stages, the collaboration with the *Research Group on Internet Technologies and Storage (GRITS)* is very effective for the acquisition of some specific competencies, but especially for the acquisition of valuable transversal competencies. The GRITS research group (specialized in areas such as future internet architectures, cybersecurity, data storage, big data, social IoT and IoT remote communications) collaborates in a series of projects, both European and national, in which students participate intending to acquire a more profound knowledge of a specific area of interest. Then, they use this knowledge to elaborate on their final undergraduate thesis. This joint work provides the student with general competencies, such as the ability to make decisions, improve their interpersonal communications, manage the suitable scheduling of tasks, prioritize and/or delegate tasks meeting delivery deadlines, perform qualified oral and written communication, among others.

Additionally, in the advanced stages, due to the close relationship of La Salle (Ramon Llull University) with companies, the university provides the opportunity of collaboration activities where these companies propose competitive challenges to students (requiring different student profiles and disciplines to be solved). These challenges reveal to postgraduates the real needs of the job market and, therefore, the competencies to be acquired, especially from the group of transversal competencies.

#### 2.2.1. Definition of Competencies for IoT Cybersecurity Learning

**Figure 2:**
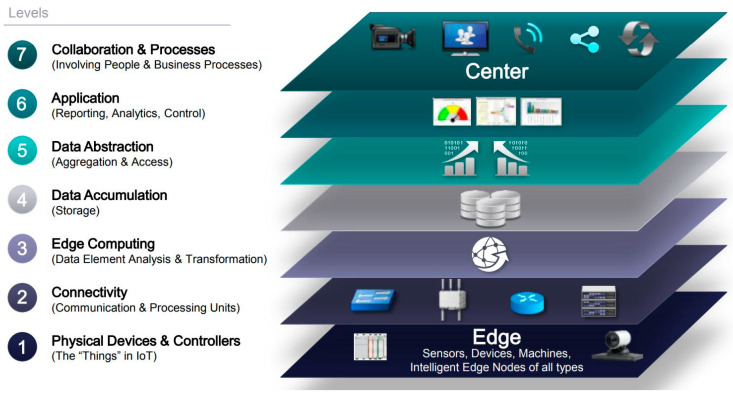
IoT World Forum Reference Model [22].

The specific competencies associated with IoT cybersecurity that the university wants students to acquire are defined taking the IoT World Forum Reference Model as a starting point [22]. This reference model is based on the premise that: *DEVICES send and receive data interacting with the NETWORK where the data is transmitted, normalized, and filtered using EDGE COMPUTING before landing in DATA STORAGE/DATABASES accessible by APPLICATIONS which process it and provide it to people who will ACT AND COLLABORATE*. As shown in Figure 2, this premise is directly transferred to a layered model based on knowledge areas. With these areas in mind, it is more affordable to achieve an effective allocation of competencies that covers the different areas.

As mentioned above, the preparation of the curricula includes the definition of the technical competencies that the students must assume. These competencies are detailed so that they are able to adapt to technological changes. This gives rise to the definition of a subset of specific competencies for IoT cybersecurity (as is done with the rest of the technologies and concepts taught in university programs). **Specific competencies** are detailed below (SC.XY indicates specific competencies as they are defined in curricula, and SC.XY-Z indicates specific competencies fine-tuned to accommodate IoT cybersecurity learning. The relevant changes are highlighted using italics):

● **SC.01:** Ability to build, exploit and manage telecommunications networks, services, processes and applications, understood as systems for capturing, transporting, representing, processing, storing, managing and presenting multimedia information, from the point of view of telematic services.
  ○ **SC.01-1**: Ability to build, exploit and manage *IoT network architectures*, services, processes and applications, understood as systems for capturing, transporting, representing, processing, storing, managing and presenting information, from the point of view of telematic services.
● **SC.02:** Ability to apply the techniques, on which the telematic networks, services and applications are based, such as management, signaling and switching systems, routing, security (cryptographic protocols, tunneling, firewalls, collection mechanisms, authentication and protection of content), traffic engineering (graph theory, queuing theory and teletraffic), pricing and reliability and quality of service, both in fixed, mobile, personal, local and long-distance environments, with different bandwidths, including telephony and data.
  ○ **SC.02-1:** Ability to apply *management, signaling, switching and routing techniques on which IoT network architectures, services and applications are based.*
  ○ **SC.02-2:** Ability to apply *security techniques to IoT network architectures, services and applications (like cryptographic protocols, tunneling, firewalls, gathering mechanisms, authentication and protection of content)*.
● **SC.03:** Ability to design network architectures and telematic services.
  ○ **SC.03-1:** Ability to design *IoT network architectures*.
● **SC.04:** Ability to program telematic, networked and distributed applications and services.
  ○ **SC.04-1:** Ability to program telematic, networked and distributed applications and services *for IoT environments*.
● **SC.05:** Conceive and develop centralized or distributed computer systems or architectures integrating hardware, software and networks.
  ○ **SC.05-1:** Conceive and develop centralized or distributed computer systems or architectures, *integrating IoT hardware, software and networks.*
● **SC.06:** Original exercise to be carried out individually and presented and defended before a university court, consisting of a project in the field of specific technologies of Telecommunications Engineering of a professional nature in which the skills acquired in the learning process are synthesized and integrated.
  ○ **SC.06-1:** Original exercise to be carried out individually and presented and defended before a university court, consisting of a project *in the field of IoT technologies,* in which the skills acquired in the learning process are synthesized and integrated.
● **SC.07**: Ability to function in contexts that provide imprecise specific information.
● **SC.08**: Ability to identify key issues that need to be addressed to solve a complex problem.
  ○ **SC.08-1:** Ability to identify key issues that need to be addressed to solve a complex problem *in IoT environments*.
● **SC.09:** Ability to design, create, develop and carry out new and innovative projects in the relevant areas of specialization.
  ○ **SC.09-1:** Ability to design, create, develop and carry out new and innovative projects in the *IoT technological area*.
● **SC.10:** Ability to integrate knowledge, deal with complexity, and formulate opinions with limited information.

As shown in the definition of the above competencies, they are defined so that different subjects or activities may fulfill them. For this reason, all subjects in one curriculum of a university program define their subset of specific competencies, adapting to the learning that is transmitted to the student. This is the exercise carried out for the acquisition of IoT cybersecurity competencies within the presented strategy.

The following table (Table 1) shows the mapping between the specific competencies and the IoT World Forum Reference Model.

Some of the reference model levels have no competencies assigned because our main learning objective is focused on network architectures that support IoT environments considering cybersecurity in communications. 

Earlier in this document, the importance that students assume some transversal competencies is explained, preparing them for the global market. **Generic competencies** are detailed below. These are included with the specific ones, although they are not directly related to IoT or cybersecurity, because a professional worker with knowledge in these topics who has no communication skills, does not know how to work in a team, is not self-critical, indecisive, and does not have the most generic skills mentioned in Section 2.1.1 and Section 2.1.2, is worthless.

● **GC.01**: Know and apply basic elements of economics and human resource management, organization and planning of projects, as well as legislation, regulation and standardization in telecommunications.
● **GC.02**: Ability to work in a multidisciplinary group and a multilingual environment, and to communicate, both in writing and orally, knowledge, procedures, results and ideas, related to telecommunications and electronics.
● **GC.03**: Know how to apply the knowledge to a job or vocation in a professional way and possess the competencies that are usually demonstrated through the elaboration and defense of arguments and the resolution of problems within the area of study.
● **GC.04:** Ability to collect and interpret relevant data (usually within ‘students’ area of study) to make judgments that include a reflection on relevant social, scientific, or ethical issues.
● **GC.05:** Transmit information, ideas, problems and solutions, to both a specialized and non-specialized audience.
● **GC.06:** Develop those learning skills necessary to undertake further studies with a high degree of autonomy.
● **GC.07:** Critical ability and intellectual defense of solutions.

These generic competencies are not assigned to the IoT World Forum Reference Model. They are competencies that are not directly related to the IoT and cybersecurity topics, but rather to the creation of valuable engineers, and consequently considering them is important.

#### 2.2.2. Learning Methodologies

The set of defined competencies is acquired through the application of different learning methodologies. For a long time, we have applied methodologies in which the students are jointly responsible for their learning. We introduce students in their own learning context so that they are more aware of the process gaining commitment, dedication, and satisfaction. Below the learning methodologies used in this pedagogical strategy are described.

● **LM.01: Laboratory Activities**. It involves the student solving a problem or making decisions using the knowledge learned in theory. Specific equipment from a laboratory or workshop is used. Laboratory tutorials, simulation exercises, field study, computer practice, company visits, or field trips are some activities that can be performed.
● **LM.02: Self-Paced Learning.** Methodologies where the student learns new content on their own, from the teacher’s guidelines or by means of didactic material designed for this purpose. Autonomous learning, self-learning, directed study, tutorials, virtual network work, individual or group work outside the classroom, or personal study are some activities than can be performed.
● **LM.03: Challenge-Based Learning.** The methodology is focused on five key points: (1) pedagogical approach that actively involves the student, (2) based on a real problem, (3) close to the student environment, (4) demand a specific solution, and (5) within the experiential learning.
● **LM.04: Project-Based Learning.** It involves the development of the subject, based on solving a project or various projects (in groups or individually) where the student discovers the concepts as they are necessary for its development.
● **LM.05: Case Method.** It consists of several stages: (1) preliminary phase: reading and study of the case for awareness of individual work, (2) phase of expression of opinions and judgments: individual reflection and the detection of descriptions of individual work, (3) contrast phase: joint analysis of the analyzed data, with work in small groups and sharing in the whole group.

#### 2.2.3. Learning Outcomes

Once the students carry out and successfully pass the activities proposed in the learning methodologies, they obtain the following learning outputs, guaranteeing the acquisition of the defined competencies:

● **LO.01:** Master the concept of network, its architecture, deployment and services for IoT environments.
● **LO.02:** Know and differentiate the concepts of transport and access network, including the most important protocols and interfaces.
● **LO.03:** Mastery of the design, configuration and implementation of the equipment that forms an IoT network architecture.
● **LO.04:** Master the analysis, diagnosis and resolution of network problems for IoT environments.
● **LO.05:** Analysis and design of security in networks for IoT systems.
● **LO.06:** Know the security technologies and their application in data systems and networks.
● **LO.07:** Have deepened in a specific subject of the area of study of the degree, applying the knowledge learned throughout it, with the ability to analyze and solve problems in an original or novel way.
● **LO.08:** Have the ability to organize and plan, searching skills and information management.
● **LO.09:** Having communicated the work performed in writing, and publicly presenting it in front of experts and non-experts in the field.
● **LO.10:** Have demonstrated the ability to conceive, design, implement, and adopt a research process of considerable scope with academic integrity.
● **LO.11:** Have demonstrated critical analysis, evaluation and synthesis of new and complex ideas.

To conclude this section, the mapping between stages, competencies, learning methodologies and learning outcomes is presented in Table 2.

In the following sections, the application of the methodologies used in each stage will be detailed, and the competencies and learning outcomes will be related according to the assessments carried out.

## 3. Acquisition of Competencies in the Early Stages

In this section, the competency acquisition in the early stages is presented through two subjects used to bring students the basic knowledge about IoT and cybersecurity. Information gathered from IEEE Internet of Things [23], ISECOM [24] and OWASP Internet of Things Project [25], among others, helps us to conform and maintain the knowledge provided to students through these subjects up to date. The subjects are ‘Networking Laboratory’ and ‘Cybersecurity’.

### 3.1. Networking Laboratory Subject Methodology

This section describes the employed methodology in the ‘Networking Laboratory’ subject. In it, students (in pairs) face a different lab each week during the semester. These students are Telematics Engineering and ICT Management undergraduate students between 20 and 23 years old. They have learnt the basics of networking in the previous courses, and this subject aims to complement their knowledge, with new skills related to advanced routing, advanced architectures like those developed for IoT environments and introduce cybersecurity concepts focusing on networking. Each of the labs focuses on a specific IoT topic, most of them related to the lower layers of the IoT Reference Model (end & network devices, network architecture, and security). Depending on the type of equipment and technologies used, labs can be deployed in one or more of the following ways: (1) physical implementation, where students use real-world devices (i.e., Palo Alto Networks Next-Generation Firewalls and Cisco Nexus 7000); (2) emulation, where students use emulating software to implement more dense and complex scenarios (i.e., Cisco VIRL [26] and GNS3 [27]); (3) in-lab virtualization, where students use the university assets to virtualize both end and network devices (i.e., VMWare ESXi and VMWare Workstation); and (4) cloud-based virtualization, where students use the Infrastructure as a Service (IaaS) resources of a cloud platform (Amazon Web Services) to configure and implement their scenarios.

Each lab consists of a theoretical explanation that students must read prior to class (**LM.02** methodology), and a few practical scenarios that must be configured and implemented during the class (**LM.01** methodology). Once students complete them, they have learnt and consolidated the concepts of a specific IoT topic in an eminently practical way. Each lab can be seen as a puzzle piece of the IoT network architecture and security, which leverages the consolidation of both theoretical and practical skills upon its completion. These puzzle pieces are combined together at the end of the semester, when students individually face a final hands-on project and a final hands-on exam. Both of them consist of a 100% practical scenario that merges different technologies, equipment, and concepts seen throughout the semester, which the student must correctly combine to make the proposed scenario work. Students prove the acquisition of the required competencies and skills upon the successful completion of both the project and the exam.

As the IoT ecosystem is continuously evolving, the subject’s labs must evolve as well to form state-of-the-art skilled engineers. In order to achieve that, during the last three academic years, 10 of the 21 labs that the subject consists of have been renewed (Table 3 shows the current lab curriculum). In addition, this course has experienced the highest impact modification, due to the recent crisis that a lot of countries have faced with the COVID-19 emergence. In Spain, all schools and universities had to close their facilities, and all lessons had to be taught virtually by using on-line tools, such as Zoom or Blackboard Collaborate. This fact precluded the continuity of the subject’s methodology as it was initially designed, because all the assets were physically placed on the university campus. Remote access to the lab’s equipment could be achieved, but it was not a feasible solution, due to the continuous change in the wiring, interconnection, and configuration of virtual and physical devices. Despite these difficulties, students had to experience no disruption in their learning process, so instructors decided to migrate all the labs of the semester to the public Cloud. By doing this, all labs had to be redesigned to meet the public Cloud infrastructure requirements and limitations, while keeping the learning skills, objectives, and topics that each lab covered intact. In total, ten labs were redesigned and migrated, and one new lab was created as an introduction to the cloud platform. The main advantage of this solution is that students can have 24/7 access to their scenarios and have total control of it.

On the contrary, before the migration to the Cloud the access was limited to the laboratory availability. Furthermore, the use of a cloud platform introduces new concepts and skills that students learn. This is very relevant if we consider that Cloud computing was the hard skill that companies needed most in 2019, according to LinkedIn data [28]. Among all the available IaaS cloud platforms, the professors chose Amazon Web Services, based on its ease of use, wide variety of configuration options and their commitment that students would not suffer any economic cost when using their platform to carry out the labs. 

The following subsections describe some of the most relevant labs carried out by students in the ‘Networking Laboratory’ subject. For each lab, a description of its contents and scenarios are given.

#### 3.1.1. Wireless Networks for IoT

In this lab, students learn how to design and configure wireless connectivity for IoT end devices.

First of all, a description and a taxonomy of different wireless technologies are presented. These protocols are classified by their coverage area, defining three main groups: PANs (Personal Area Networks), LANs (Local Area Networks) and WANs (Wide Area Networks). Some of the most relevant protocols for IoT presented are Zigbee, Sigfox, 6LoWPAN, LoraWAN, NB-IoT, 802.11, LTE, 5G, and Bluetooth, among others. From all these protocols, a more detailed explanation is given for the 802.11 standard, which will be used for the practical scenarios.

The practical scenarios focus on two approaches for deploying a WLAN (Wireless LAN): using a physical wireless controller to manage the Access Points (APs) (with Netgear equipment) or using a cloud-based wireless controller to control the APs (with Cisco Meraki equipment). 

**Figure 3:**
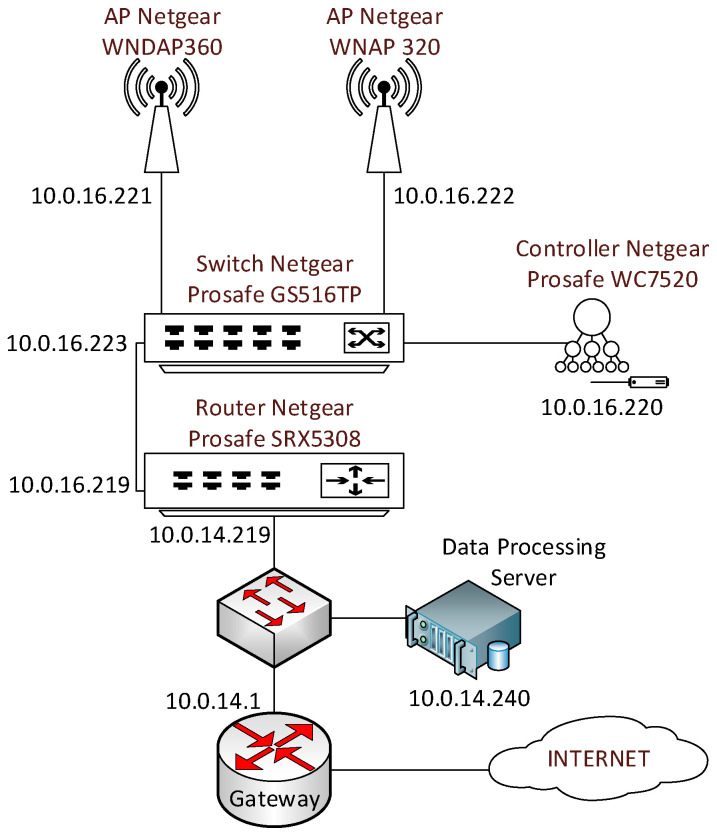
Physical Wireless Controller Lab.

The scenario that students must deploy with the physical wireless controller is shown in Figure 3. In this scenario, students learn how to configure different SSIDs (Service Set Identifiers) and VLANs (Virtual LANs), each of them with a unique security profile. Additionally, management access is granted to administrators through a captive portal, where every administrator must authenticate themself with their own user credentials. Students successfully complete this scenario when they show the instructor that the whole network is working, and the end devices can communicate between them and send data to the data processing server.

**Figure 4:**
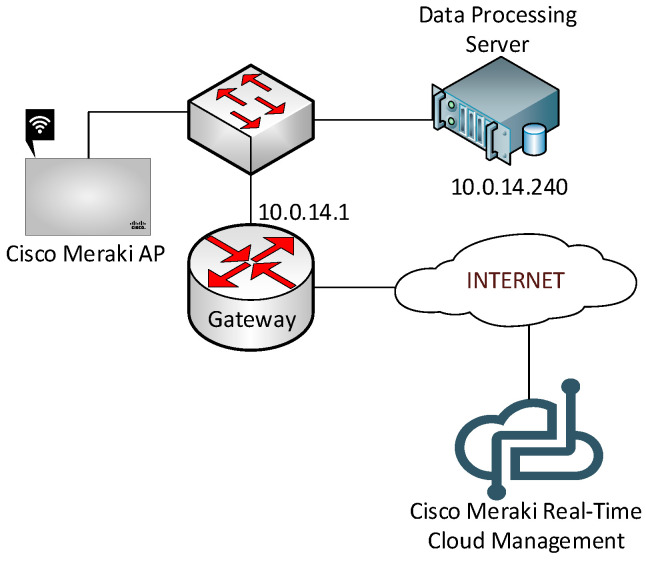
Cloud-based Wireless Controller Lab.

On the other hand, there is the cloud-based wireless controller scenario with the Cisco Meraki AP. Figure 4 shows the topology for it. With Meraki Cloud Management, students learn how to set different types of wireless profiles, each one with its own security and access settings. Students have to accomplish the same requirements as in the previous scenario to pass this one.

**Figure 5:**
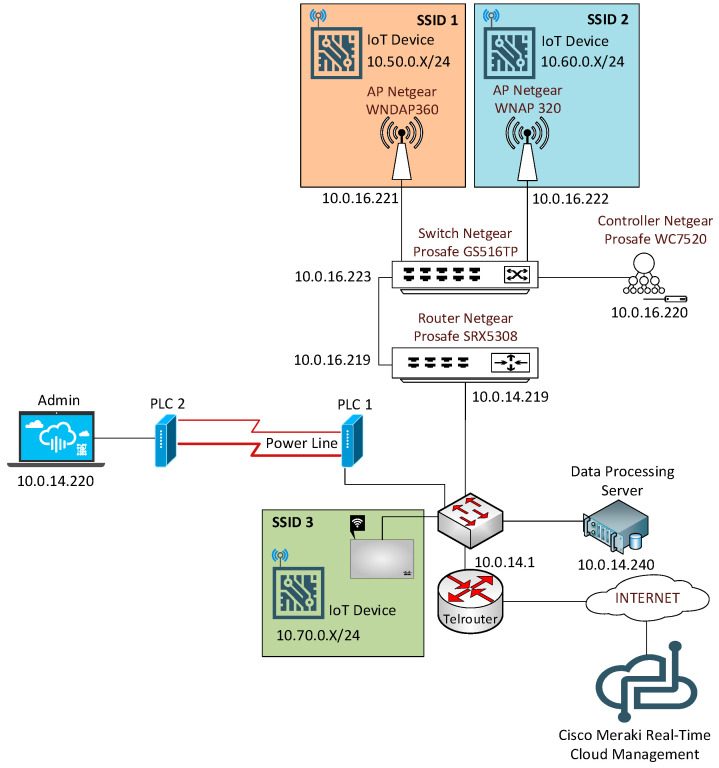
Final WLAN Lab.

At last, there is a final scenario which merges both topologies and asks students to successfully communicate end devices connected to the Meraki AP with the devices connected to the Netgear APs, as shown in Figure 5. Additionally, Power Line Communications is introduced to interconnect two gateways without the need for wired UTP/STP infrastructure. When all devices get full connectivity with the rest of the scenario, students pass the lab.

#### 3.1.2. Palo Alto Advanced Firewalling

**Figure 6:**
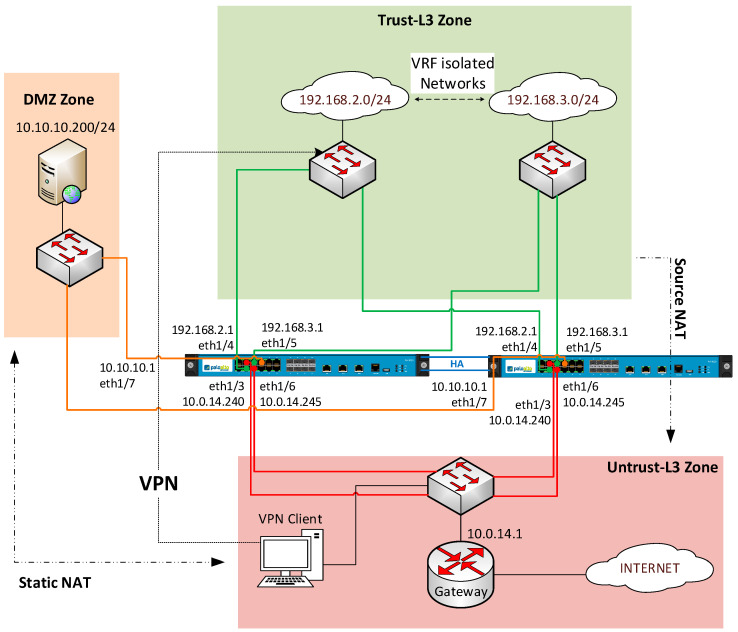
Palo Alto Advanced Lab Scenario.

This lab focuses on the advanced features that Palo Alto firewalls can offer to control and monitor IoT network architectures. The scenarios are composed of three main zones: the External Zone (Internet), where devices must be protected from; the DMZ Zone, where data processing servers are placed; and the Internal Zone, where the IoT devices are connected (see Figure 6). The basic firewalling concepts are already assimilated from previous labs, so this one focuses on introducing new and advanced ones.

First, the student must be able to interconnect the three zones together, while performing NAT (Network Address Translation). This helps to hide the internal devices and sensors from the outside, so external users cannot know which is the internal topology and the real addressing from these devices. Once inter-zone connection and NAT are configured, students have to apply policies to control which applications and services are permitted (i.e., from the external networks administrators must be able to access the servers for monitoring purposes, while only the end-devices from the internal networks can send data to those servers). In addition, threat detection must be configured to analyze and counteract against possible malicious attacks from both external users and bogus internal devices. Furthermore, SSL inspection policies are added to decrypt the traffic that the firewall must inspect in order to apply policies correctly. When students successfully configure all these requirements, they can pass to the next phase.

After that, students are introduced to the concept of High Availability (HA) and how to configure it on the firewall. They learn that, by implementing HA, they remove a single point of failure at the edge of the IoT network and minimize the risks of a critical failure. Once it is configured and its functioning is verified, students move to the next phase, where the concept of Virtual Routing and Forwarding (VRF) is presented. VRF is very useful when the same firewall (or group of firewalls) must connect to independent IoT networks serving different purposes. As these networks do not need to know each other, they must be able to use the same private addressing. Students configure VRF in the firewalls, so these networks are isolated, and the same IPv4 private addresses can be used, learning the main advantages of this technology. To move to the next phase, students must prove that both internal networks can communicate with the DMZ and External zones, but they do not reach each other.

Finally, students have to include the use of a VPN into the scenario. By implementing a VPN, they gain access to internal resources from the external network, and they learn the main advantage of it, which is that there is no need to expose IoT devices or services to the public to access them, minimizing the related potential security risks. 

#### 3.1.3. Introduction to Amazon Web Services

As has been mentioned earlier, an important task has been conducted to adapt the’ Networking Laboratory’ subject, due to the health alert caused by COVID-19. The new skills that students gain, learning how to work on a cloud platform, may help them acquiring relevant knowledge for hosting and managing IoT application instances in Cloud environments.

This lab is a key component for all the semester, as students learn how to set up the AWS platform that they have to use for the rest of the labs. Each student has its own account to access AWS, where they can configure their own scenarios. This fact gives students a lot of flexibility, so they can choose when to complete the required activities, and there are not time, availability or resource limitations.

### 3.2. Cybersecurity Subject Methodology

This section describes the design and implementation of the learning platform for IoT security training that was developed at La Salle (Ramon Llull University), our learning platform that helps IoT security trainers to teach knowledge on security testing and auditing IoT architectures by using hands-on technological training. The IoT learning platform is composed of several parts, including the testbed, the eLearning platform, the management interface, the learning material, and the proposal of challenges offered to students.

The testbed has been designed to replicate real infrastructures where students find and experiment with several and diverse IoT platforms, Operating Systems (OS), network topologies and protection equipment, all organized in several and different scenarios. The students use this testbed to implement the labs proposed in the different lessons of the Learning Materials, where a specific scenario is designed for each of the lessons, to accomplish the defined objectives.

**Figure 7:**
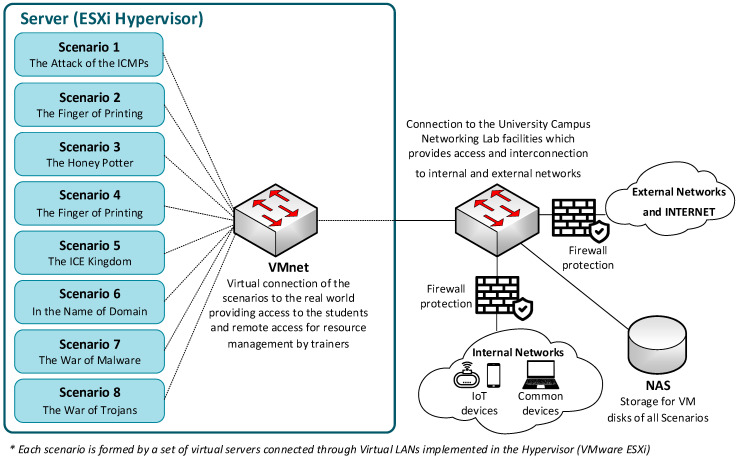
Learning platform for IoT security training: Testbed implementation.

The design and implementation of the testbed are based on the concepts of virtualization and cloud computing on the University Campus’ assets, with three main parts: (1) network virtualization based on Virtual LANs concept to implement the different scenarios, providing the required flexibility to change the topology just by configuration, (2) server virtualization built in a server farm running a hypervisor that supports the needed Virtual Machines (VM), and (3) storage virtualization by using a Network Attached Storage (NAS) to centralize all the VM disks in a single place, allowing the movement of VMs between servers to accomplish the requirements of high availability and flexibility (see Figure 7).

Our students use the management interface, or front-end, to log into the environment and check the different scenarios that they must implement. Once they are logged in, an access firewall allows them to access the corresponding scenario of the testbed. The access to the testbed is supervised by the instructor, who controls the activity of each student, using the information obtained by a monitoring system. Using this information, the trainer can certify whether each student has accomplished the objectives for each lesson or not. The objectives defined for each scenario are specified in Table 4.

The teaching process of the ‘Cybersecurity’ subject carried out in the second semester of third academic year, is divided into the following course development phases:

● **Training**: In the first part of the course, students are trained throughout all the lessons described previously using the “student’s workbook”, composed by around forty different labs (**LM.01** and **LM.02** learning methodologies). These labs are performed in pairs of students from Telematics and Computer Engineering undergraduate programs (between 20 and 23 years old). They have learnt the basics of networking, operating systems, and programming languages in the previous courses. Table A1 in Appendix A shows the objectives of the labs performed by using step-by-step learning techniques, methods and tools that will be applied for auditing and protecting an IoT network environment. With the knowledge that they obtain, they are then ready to face up to the second and most challenging part of the course.
● **Challenge**: After the initial training period, students must demonstrate the knowledge and skills acquired. In order to motivate them and get the best of them, our proposal for the development of the security course is to challenge them in a kind of competition or hacking contest. Based on the Project Based Learning (PBL) methodology (**LM.04**), we propose them a Challenge Based Learning (CBL) activity (**LM.03**), where they must compete in groups to demonstrate who the best ethical hackers of the year are.
● **Presentations**: Before finishing the course, students must present their results, not only to the rest of the groups, but also to trainers and, most importantly, to IoT security experts from external companies. Across this activity, they get in contact with real enterprises, and these corporations could evaluate new candidates to hire.

At the end, the obtained results have been exposed. Moreover, motivated, skilled and valued engineers are obtained through the security course developed. The aim of the ‘Cybersecurity’ subject is to complement students’ knowledge, with new skills related to cybersecurity and ethical hacking to be applied afterwards in IoT and non-IoT environments, and provide a nearby experience to the cybersecurity labor market (also assuring the acquisition of some soft skills). Nevertheless, the IoT platform is in constant evolution and improvement, where not only the students in their last year contribute to its development (via activities like final thesis elaboration), but the students enrolled in the security course also participate, increasing the number of learning materials and resources available on it.

For this subject, no examples are presented, because the labs are made up of a large set of exercises that follow the same methodology (**LM.01** and **LM.02**) to meet the objectives defined in Table 5. All laboratories have great importance within the field of cybersecurity applied to IoT architectures; therefore, each one would be remarkable.

On the other hand, it should be noted that the hacking contest carried represents an incentive challenge for students due to its methodology (**LM.03**) and implementation.

### 3.3. Competencies Acquired by Students and Learning Outcomes

The competencies and learning outcomes defined for early stages are distributed between the subjects, as shown in Table 5.

## 4. Acquisition of Competencies in the Medium Stages

In this section, the competency acquisition in the medium stages is presented through the students’ collaboration and the final thesis elaboration in the Group of Research in Internet Technologies and Storage.

### 4.1. Methodology

The *Group of Research in Internet Technologies and Storage* (GRITS) is a group of academic researchers from different disciplines who focus on addressing the following technological areas:

● Future Network Architectures
  ○ Next Generation Networks (Industry 4.0, eHealth, SmartHome, SmartCities and Smart Grids environments)
  ○ SDN/NFV/DevOps (Service Composition, Services Definition, OSGi, composition orchestrator, among others)
  ○ Transport (fairness, wireless, in-vehicle networks, among others)
  ○ Web of things (social objects, semantic representations, behaviour/state definition, AI, among others)
  ○ Cybersecurity (for IoT environments, for critical infrastructure networks, among others)
● Cloud computing
  ○ ○ Cluster computing
  ○ High-performance computing
  ○ Grid computing
  ○ Parallel computing
● Remote IoT
  ○ NVIS systems/remote sensing
  ○ Innovative types of antennas and compact antennas

The acquisition and renewal of knowledge in these areas is carried out by performing European and national projects, that are usually done jointly with other academic institutions and companies. In this way, the knowledge acquired is subsequently transferred to the different undergraduate and postgraduate programs and, therefore, to university students. In addition, two types of collaborations are carried out with students, to bring them closer to the world of research and to allow them to participate in realistic projects: (1) performing the final thesis of under- or postgraduate programs within the research group and (2) collaborating with the research group to increase and focus student learning. In fact, many students begin collaborating in the early stages, and end their collaboration by carrying out the final thesis on that topic in which they have specialized for at least one year (students between 20 and 24 years old, depending on whether they are starting a collaboration or performing the final thesis).

These collaborations are focused on helping students to:

● Learn the basic research abilities so that they carry out the correct execution of the required work.
● Learn to differentiate valid sources of knowledge to support coherent work.
● Learn to transmit and transfer knowledge through the elaboration of documentation.
● Learn to express their work and ideas orally by periodically presenting their work progress.
● Learn to focus their work on the topic of interest.

The starting point for achieving these objectives is based on a specific topic related to one research group’s technological area and which is also of interest to the student. The student must solve a problem (usually related to an ongoing European or national project), where he/she must use that topic or topics of interest. Typically, the student performs both individual and group tasks to improve soft skills. A Project-Based Learning methodology (**LM.04**) is applied throughout the process (and **LM.02**, given that guides and materials are provided so that the student can learn some of the needed concepts on their own). Everything is done through continuous tutoring that controls the evolution of the student and helps this student to improve throughout the process.

In the following subsections, a couple of examples are provided to see the type of activities performed by our students.

#### 4.1.1. Undergraduate Program Final Thesis

Taking advantage of the knowledge of the engineers who are part of GRITS, the supervision of a final thesis entitled “IoT cryptography schemes comparison” is carried out.

The main issue addressed in this thesis is the fact that the sensors/devices used in IoT networks are small elements; they will use various Operating Systems, CPU types, memory, etc. In addition, many of these units will be (or are) very cheap, with a single mode of operation and a basic network connection that does not have the power, storage capacity, computational capacity or memory to support the current encryption protocols. In order to overcome this problem, new schemes capable of running on IoT devices, based on current encryption algorithms, must be achieved.

The main objective of the thesis is to compare different solutions and draw conclusions, on which one best adapts to the current IoT devices’ limitations. To meet this main goal, several phases are performed in the thesis:

● Learn about current cryptographic schemas and their functioning (ciphering, deciphering, etc.). A state of the art on cryptography schemas is carried out comparing Symmetric Key Cryptography (studying block ciphers like CLEFIA and stream cyphers like MICKEY v2) and Asymmetric Key Cryptography (studying hash functions like SHA-3 and KECCAK, and Elliptic Curve Cryptography). Additionally, MATLAB simulations of these schemas are performed to learn which cryptographic algorithm seems the most suitable for IoT devices (focusing on performance parameters).
● Perform a fair comparison in a well-defined framework that would help to determine which schema is the best under the following premises:
  ○ Computational cost.
  ○ Data transmission rate.
  ○ Battery cost.

A set of probes is implemented to test cryptographic algorithms functionality in IoT devices performing various transmissions of encrypted data. The devices used are Zolertia Z1 mote and Sky mote with Contiki OS (one of the most frequently used IoT OS focused on cryptography). The motes are implemented in the Cooja simulator. The data messages sent are encrypted using the AES standard (an encryption standard that is successfully emulated in various platforms/motes). This simulation shows, under the same scenario and algorithm usage, which case suits the IoT world best, through a well-defined closed framework (in terms of computational cost, data transmission rate, and power consumption).

● Enhance student’s ability to research information, filter it, understand it and apply it. Specifically, from a scientific point of view.
● Learn to draw valuable conclusions from the performed work. For this thesis, the student concludes that current cryptographic algorithms are ready and suitable to use in IoT devices, in terms of performance. Each application and device will have its own limitations, so it is complicated to find a specific algorithm that will ensure correct performance, while fulfilling all the limitations.

#### 4.1.2. Student Collaboration in Research Groups

The GRITS research group is carrying out the *SmartCampus* project, which consists of creating a proof of concept of an architecture that will use IoT and Cloud/Fog computing technologies for the comfort and energy efficiency measurement, control, and visualization. The project will show the feasibility of its implementation throughout the Barcelona university campus, to provide various benefits on a day-to-day basis, for students, teachers, and university staff. An architecture like this must be scalable and robust. Additionally, it must support various communication protocols and ensure the transmissions of the messages that are sent. 

The *SmartCampus* project aims to design a proof of concept to (1) store heterogeneous data in one (or several) clouds, using the Social IoT paradigm, (2) offer a single access point for the different services of the *SmartCampus* Barcelona (Smart Lightning, Climate Satisfaction, Noise/Heat Map, Virtual Assistant, Active Listening, People Safety, Smart Parking, etc.) can access the collected data, and (3) offer a holistic and centralized view of events that are being monitored on campus. In the future, it will serve as a testbed for a Facility Manager of the Barcelona university campus.

In this project, the collaboration with students (regarding the security part) aims to present a first approach to the security of internal communications between the IoT devices that make up this *SmartCampus* architecture. Accordingly, different phases are carried out:

● **Approach to key IoT challenges, such as interoperability and security**, necessary in the design of any IoT network. These characteristics and how to implement them are studied.
● **Search for interoperability solutions** between communication protocols. Different approaches are found in the use of IoT Gateways to address this challenge: (1) One Gateway–Various protocols, (2) One Gateway–One protocol, and (3) a merging of the two previous alternatives.
● **Search for security solutions** for communications. An analysis of the application layer and M2M (machine-to-machine) protocols is performed, as they are considered a key component for communication between IoT devices and their applications. The MQTT (Message Queue Telemetry Transport) protocol is chosen because it meets the characteristics of being light, flexible, and easy to implement, in addition to being a very widespread protocol in applications such as healthcare, energy, and social networking.
● **Make a proposal for a secure communications system**. MQTT is chosen to encrypt the data using asymmetric AES encryption (128 or 256) in CBC (Cipher-Block Chaining) mode. An intermediate layer of security is incorporated within each of the MQTT PUBLISH messages (the payload of each message is encrypted). This is an easy implementation solution, which does not require many resources, and serves as a test to apply the same idea to other protocols, such as CoAP. Implementation with the One Gateway–One protocol approach adapts to the current vision of the *SmartCampus* (it is not contemplated to have several M2M communication protocols in the first phases of the project).
● **Implement the proposal**. Implement a set of tests to see the feasibility of the proposal. Two possible implementations are proposed: Middleware-based implementation and client-based client. The tests are performed for two different scenarios: (1) device-to-device communication, with a C ++ based client implemented in an Arduino MEGA and information encrypted with AES128, (2) communication between internal network and external network, with a Java SDK 1.8 client-based and information encrypted with AES 128.
● **Extract conclusions on the work done**. Students draw their own conclusions. This proposal presents an MQTT-based solution for communications between IoT devices. This protocol is adapted to the needs of the *SmartCampus*, but it has certain security limitations that must be overcome. So, a secure communications solution based on AES encryption mechanisms is proposed, and the feasibility of this solution is tested in a test environment.

### 4.2. Competencies Acquired by Students and Learning Outcomes

The competencies and learning outcomes defined for medium stages are distributed between the different activities performed in this stage, as shown in Table 6.

## 5. Acquisition of Competencies in the Advanced Stages

In this section, the competency acquisition in the advanced stages is presented through real case challenges from companies.

### 5.1. Methodology

According to Section 2, the advanced stages (postgraduate programs) of the pedagogical strategy pursue the interaction between students of different disciplines, as it happens in the world outside the university, facing real problems that companies share. The students are mainly Telecommunications or Computer Engineers between 25 and 35 years old (most of them). They may come seeking to master an area of knowledge studied during their undergraduate program training, or recycle themselves or refocus their career. For example, we have economists, mathematicians and physicists interested in joining the Big Data postgraduate. Additionally, another profile of students may be those with occupational training and years of experience, but without higher education training. Furthermore, due to the interdisciplinary nature of the current research master thesis, different approaches to work amongst multidisciplinary stakeholders will be identified in advanced stages. Our university developed the Master as a Service (MaaS) initiative as an attempt to reach this strategic objective. In [30,31], examples of previous work conducted and related to the MaaS inititive for big data topics are presented.

**Figure 8:**
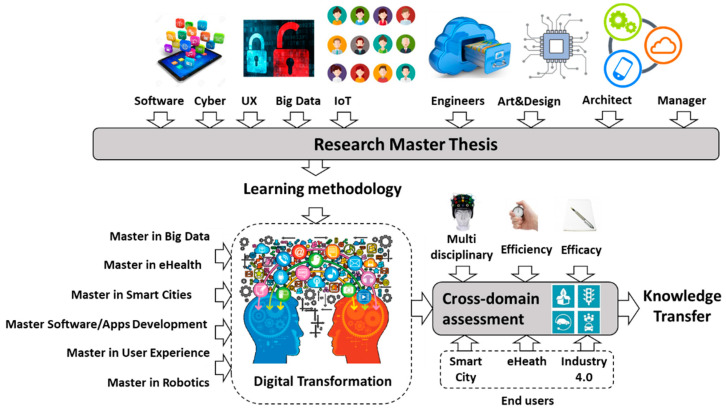
MaaS Conceptualization.

Currently, this type of interdisciplinary projects facilitated by the collaborating companies requires the involvement of different engineering profiles, since the research master thesis involves more than one topic (Software, Cybersecurity, Big Data, Internet of Things, User Experience, Business, Innovation, Architecture, etc.) within the field of engineering. Carrying out transversal projects through the different university masters by using a specific methodology for the development of a research master thesis is proposed, as well as the acquisition of the competencies established within this framework. The goal of this initiative is to provide an enriching work environment for the development of large interdisciplinary projects in the context of a real project proposed by leading companies in Innovation and Information and Communication Technologies (ICT) (see Figure 8).

Each of the individual tasks in each phase of the interdisciplinary master thesis requires its own research to provide the expected outcomes. However, some common methodological approaches are applied. The most relevant are:

● An intensive standardization work will be performed, using terminology and controlled vocabularies that will help for the interoperability of tasks and stakeholders.
● An agreement on the participant’s goals and expectations, providing a partner/user-centered approach.
● Assignation to different student groups for execution and for supervision of each task.
● Collection of regular information significant for the enhancement of the design specifications and the possible long-term implementation plan.
● Ensure and provide the most effective and reliable communication and monitoring process for each business case.

The interdisciplinary projects proposed by companies in the MaaS initiative are affordably implementing Challenge-Based Learning (**LM.03**) and the Case Method (**LM.05**) methodologies. Students must face a real problem, and propose an agreed solution with colleagues from the assigned multidisciplinary group. 

#### An Example of a MaaS Initiative: SPRINT 4.0

SPRINT 4.0, “Strategic Partnership for Industry 4.0” (2018–2020) is a European Project that works on filling the existing knowledge gap training university students on Industry 4.0 topics.

The project aims to achieve new skills and competencies for students, on one side directly related to the Industry 4.0 domain (thus enhancing the quality of their knowledge), and on the other side, supporting them in acquiring key competencies, such as entrepreneurship (or intrapreneurship), to foster their employability and professional development. Entrepreneurial skills and an entrepreneurial mindset are necessary for successfully working in the new paradigm, as redesigning an enterprise following a 4.0 approach means also being ready to reshape the traditional business model of a company. For such a reason, teaching entre/intrapreneurship methods and tools is a key asset that cannot be separated from the technology-based training.

**Figure 9:**
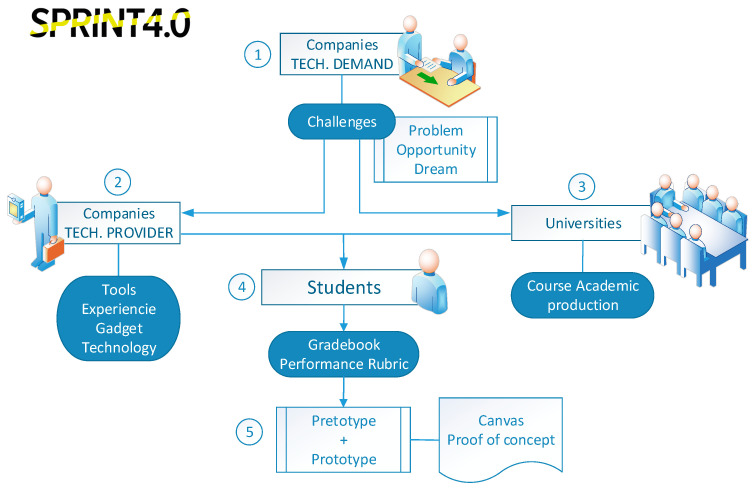
SPRINT4.0.

Figure 9 shows how challenges and proposals are structured in SPRINT 4.0. It starts with the identification of the possible problems that companies are experiencing, opportunities detected or future purposes. From these conceptions, several challenges arose. Together, with the universities and tech providers, it is decided which concepts are necessary to be taught to their students in order to prepare them for the real case resolution. Then, companies provide a challenge that students must address, and for which they can use specific technological providers (also participating in the initiative). The solution provided by the students is analyzed to decide its viability, and then is pre- or prototyped, generating a proof of concept.

In the academic year 2018–2019, Selettra was the company that proposed the challenge. Selettra possesses over 400 production tools that can be used inside and outside the factory by collaborators. These tools, with the size of a shoe, are very expensive and critical for the production. At the moment, it is challenging to manage them, since it is hard to know if they are still inside the factory or not, because collaborators take them without any kind of control. Furthermore, nowadays, tools’ maintenance is done ‘on-demand’, which makes it very inefficient. 

The company claims that knowing the tools’ position and status would improve the precision of production and shipment planning as well as customer service. Moreover, having a model of predictive maintenance would decrease maintenance costs and increase production efficiency and quality. Therefore, Selettra proposes the implementation of a solution that covers these needs and helps them meet the mentioned impacts.

To solve the mentioned problem, several interdisciplinary groups of students proposed different solutions. The winner solution was the use of a Real-Time Location System (RTLS). Using RTLS to locate assets and improve efficiency can be a financial and compliance lifesaver for today’s organizations. Electronically tracking assets allows organizations to better manage what they have, streamline staff efficiencies, and reduce loss, which in turn improves customer satisfaction and bottom-line savings.

**Figure 10:**
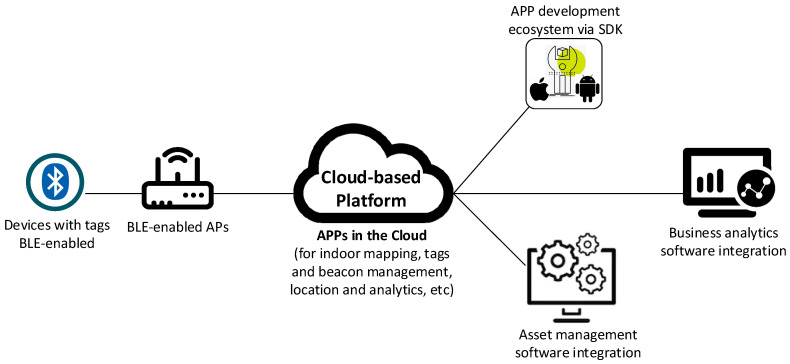
Asset Tracking.

This solution uses a management and tracking software and pairs Bluetooth Low Energy (BLE)-enabled tags with BLE-enabled wireless access points (APs). These tags are small, low power wireless transmitters that broadcast 2.4 GHz radio signals at regular intervals. These signals are heard by BLE-enabled access points, which act as observers, and provide the sensory network. Tracking information is then sent to the management cloud-based platform, where location mapping allows for each asset to be viewed in its actual location. A diagram of the mentioned architecture can be found in Figure 10.

To complement the mentioned system, the use of a box was also proposed, that reads the information of the tools placed inside, together with a QR code reader as the checkout method for collaborators. Before exiting the site, in order to open the door, collaborators must place the tools inside the checkout box, and scan their unique QR code. The system then registers that these tools are outside the factory at the collaborators’ site. When a collaborator wants to return a specific tool, the same process must be followed.

In order to predict the maintenance of tools, a functionality that allows setting alarms, when a certain percentage of the Mean Time Between Failures (MTBF) of the tools has passed, must be implemented in the Management and Tracking Platform. Furthermore, factory operators must register every time a tool is broken before the expected time, so that the percentage can be adjusted more precisely.

There is no feasible solution without taking security into account. To secure the presented system, security has been considered in several parts. First of all, security has been integrated with the system inside the company by placing a Next-Generation Firewall at the edge of the Enterprise Network, thus providing perimeter security. Then, to transmit the data to the Management and Tracking Platform over the Internet, it uses the protocol TLS (Transport Layer Security) with the algorithm DHE-RSA for key exchange, AES CCM-256 for data encryption and AEAD for data integrity. Therefore, it will be running TLS over HTTP (HTTPS) for a secure data exchange. To access the Cloud, the App will have a Role-Based Access Control, so that only authorized personnel can access the private data. Finally, to make sure that exclusively authorized collaborators take out tools from the factory, collaborators will authenticate using their personalized username and password, to be able to log in to the customer mobile app.

This students’ proposal implementing IoT cybersecurity topics was possible by combining the knowledge that each student obtained in the undergraduate and postgraduate studies (the group was composed of telecommunications, computer, and network engineer profiles).

### 5.2. Competencies Acquired by Students and Learning Outcomes

The competencies and learning outcomes defined for advanced stages are defined in Table 2. As we only have one activity in this stage of the pedagogical strategy, they have been defined earlier in the document.

Furthermore, as each challenge of the MaaS initiative is focused on specific topics, the additional learning outcomes are defined to be aligned to the objective of the challenge. The additional learning outcomes of SPRINT 4.0 are:

● Understand and use the technologies used to process big data.
● Interpret the security risks associated with Industry 4.0 and apply the corresponding countermeasures to minimize these risks.
● Categorize the technologies used for the administration of Industry 4.0 management systems.
● Identify and analyze the requirements and needs of challenges posed by companies in the Industry 4.0 sector, and propose different solutions to these challenges, as would be done in real projects.

It should be pointed out that activities proposed in the MaaS initiative are focused on providing the knowledge to apply and work with technology, instead of adding new basic concepts. Therefore, the MaaS initiative seeks that students will be able to recognize and practice the skills needed in the real world.

## 6. Result Analysis and Discussion

In this section, the results obtained by the students and the feedback provided by them are analyzed to check if the pedagogical strategy proposed is suitable and to collect information needed for continuous improvement of the methodology applied.

### 6.1. Students Results Analysis

A substantial metric to evaluate the pedagogical strategy is the grades of the students. These grades are obtained through assessments performed using learning methodologies explained in this paper. The application of these methodologies helps students to acquire the desired competencies and therefore, successfully passing subjects implies their acquisition. All the evaluable activities carried out within a subject are graded from 0 to 10, passing them if a mark equal to or greater than 5 is obtained. Additionally, a subject is successfully passed if its final grade is equal to, or greater than, 5. 

For the early learning stages, we will analyze the academic results of the ‘Networking Laboratory’ and ‘Cybersecurity’ subjects.

**Figure 11:**
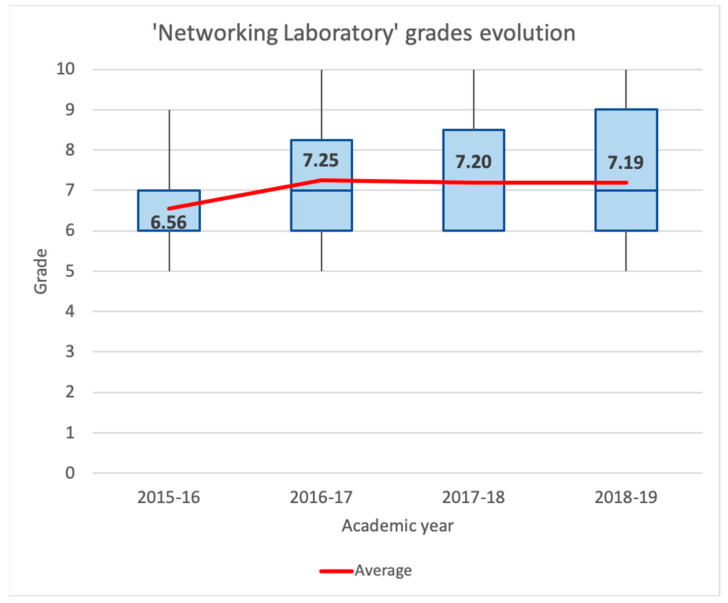
Evolution of academic results of ‘Networking Laboratory’ subject.

Figure 11 shows a box plot for the ‘Networking Laboratory’ marks’ evolution from the academic years 2015–2016 to 2018–2019. The figure also displays the average grade for each one. Throughout all academic years, the minimum grade is 5, except for 2017–2018, where the worst mark was a 6. While in the academic year 2015–2016, 75% of grades were equal or below 7, students achieve remarkably better results in the following years, with 75% of marks being equal or greater than 6. The best academic year in these parameters is 2018–2019, with 50% of grades between 7 and 9 and 25% between 9 and 10. Furthermore, the average grade increases by 10.52% from 2015–2016 (6.56) to 2016–2017 (7.25) and maintains similar numbers (7.20 and 7.19) during the following academic years. This increase in the average grade and the redistribution of marks to higher numbers coincide with the introduction of the pedagogical strategy described in this paper to the classrooms, beginning in the 2016–2017 academic year.

**Figure 12:**
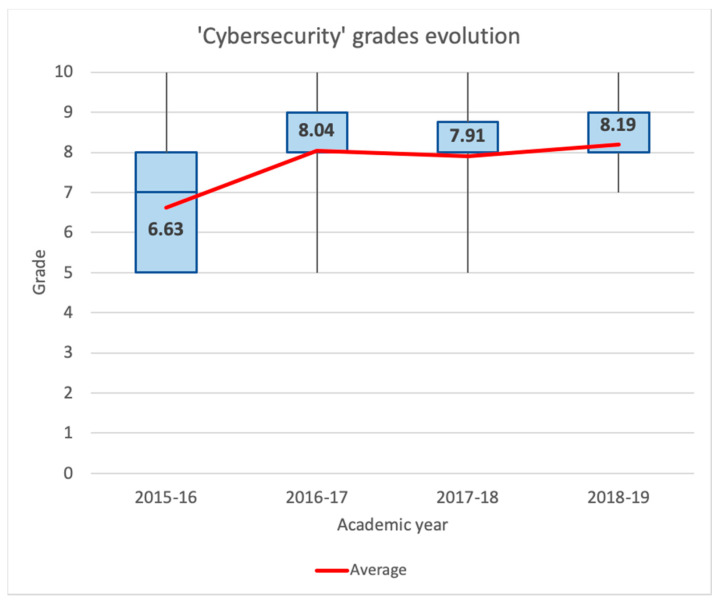
Evolution of academic results of ‘Cybersecurity’ subject.

Figure 12 shows the same statistics for the ‘Cybersecurity’ subject. At first sight, we can observe similar results. We can see that the minimum grade is always 5, except for the last year, which is 7. Students achieve better results beginning in the academic year 2016–2017, which is the first time that lecturers introduced the described active learning methodologies. Observing the last three years, it is remarkable that 75% of the marks were equal to or greater than 8. This fact is a substantial change compared to the academic year 2015–2016, when 75% of grades were equal to or below 8. Moreover, during the last three years, the average grade was between 7.91 and 8.19, while in the academic year 2015–2016, the average was 6.63. If we compare the year 2015–2016 with the year 2016–2017, the average grade increased by 21.27%.

Another essential aspect of analyzing the new active learning methodology is to consider the evaluations made by stakeholders external to the university. During the early learning stages, companies have the opportunity to evaluate the final project done in the ‘Cybersecurity’ subject. From the 2015–2016 academic year, to the 2018–2019 academic year, 29 companies participated in this activity (some of them are big companies which are globally recognized, like KPMG, PwC, Deloitte and Ernst & Young, and IT or cybersecurity consulting companies, like Accenture, GMV, Be12, Blueliv, and Brightsight, among others). These companies perform individual interviews to evaluate technical skills (proven technical abilities to work in the cybersecurity labor market through the results obtained and the documentation presented in the final project), and soft skills (communication skills, empathy, ability to teamwork, language used, presence, among others).

**Figure 13:**
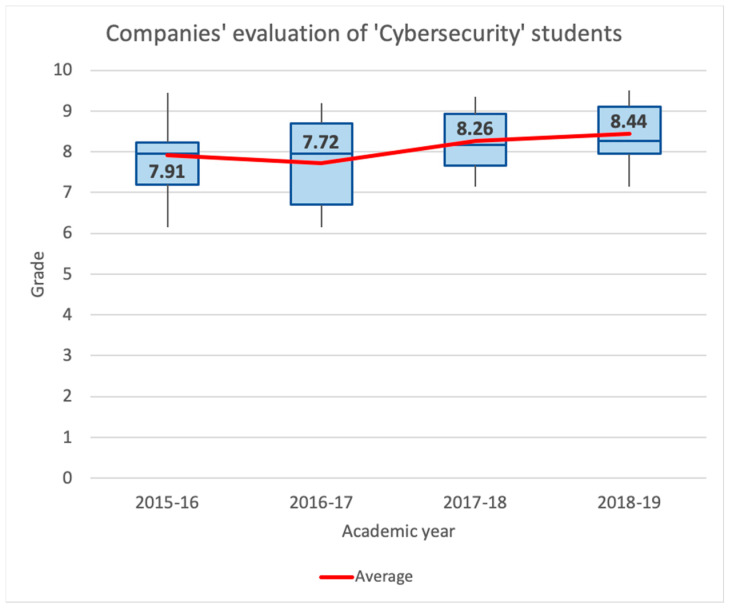
Evolution of companies’ evaluation of ‘Cybersecurity’ students.

Figure 13 shows the results of these evaluations. First of all, it is interesting to note that the obtained marks are good over the course of all the academic years, since the minimum grade is always higher than 6.1, and the average is above 7.7. In addition, we see that over the last three years in which active methodologies were introduced into the subject, the evaluations’ trend is rising. During the last two years, students obtained, in general, better marks from the companies, so that both the mean and median values of them are greater than 8. Moreover, in the 2018–2019 academic year, nearly 75% of students got a grade above 8.

These results fit with the feedback that companies gave to the professors of the subject. They showed immense satisfaction, both with the activity and the skills and competencies acquired by the students, and their desire to repeat the experience in the following years.

Regarding medium stages, 12 undergraduate program final theses about IoT and cybersecurity topics have been presented since 2016. These final theses were focused on auditing methodologies applied to smart environments, monitoring and management of IoT environments, applying blockchain to social IoT, firewalling and security event management, and improving IoT security training platform of ‘Cybersecurity’ subject, with new labs and updated scenarios, among others. Some projects were performed in collaboration with the GRITS research group, but not the totality of them. 

The evaluation of the project presented is based on the following criteria (the mentor of the project and a tribunal of three people evaluate each criterion):

● Have studied, in depth, a specific subject of the area of study of the degree, applying the knowledge learned throughout it, with the ability to analyze and solve problems. The evaluation of this criterion depends on the originality of the way the student addresses the problem and how the work is orally presented.
● Have the ability to organize and plan, search and manage information skills. The evaluation of this criterion depends on the tracking performed by the mentor and the project report presented.
● Have communicated the work performed in writing and publicly presenting it in front of experts and non-experts in the field. The evaluation of this criterion depends on the project report presented and how the work is orally presented.

All students obtained marks above 8.5, except one who obtained a 7 three of them graduated with honors (the highest mark), and three other students with a mark of 10. The average grade of all these final theses is 9.25. These results are those expected according to the effort done by every student. Some topics addressed are quite novel, and students now have more specific knowledge about IoT and cybersecurity.

Finally, regarding the advanced stages and the MaaS initiative, the most relevant activity done in the last three years related to IoT and cybersecurity is the mentioned example of the SPRINT 4.0. Other MaaS initiatives have been performed, but these topics were addressed separately. So, we consider that the results of the SPRINT 4.0 are the important ones to be shown.

**Figure 14:**
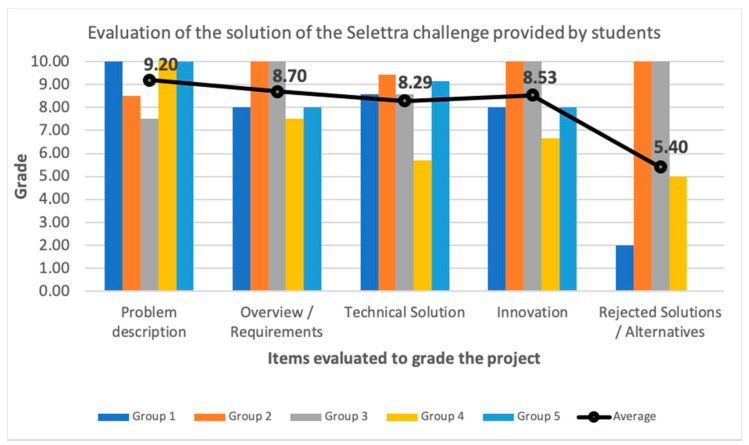
Evaluation of the results of the Selettra challenge.

The final evaluation of the SPRINT 4.0 course was the presentation of the solution of the Selettra challenge. To solve this challenge, the 21 students of different postgraduate programs (Big Data, Cybersecurity and Smart Cities) participating in the SPRINT 4.0 course were divided into five groups, mixing these profiles inside each group. The following aspects (Table 7) were taken into account, in order to evaluate the learning outcomes (see Section 5.2):

The grades obtained for the items evaluated are presented in Figure 14, differentiated by groups. Moreover, the average for each item is shown. All the groups presented a correct description of the problem and, especially, three of them fully understood and explained the Selettra challenge, obtaining an average mark of 9.20. There was also a good perception of the requirements that the solution to be provided by the students must meet, with an average grade of 8.70. Each group suggested an appropriate technical solution, explaining the technologies, systems and resources necessary to carry it out (average mark of 8.29). Only one group did not elaborate on the technical explanation of their solution and therefore obtained a regular grade of 5.70. The proposed solutions have a high degree of innovation (average mark of 8.53), using new generation technologies and advanced systems. Finally, only two groups made an appropriate analysis of possible solutions, and presented the pros and cons of each one, justifying their final decision, causing a low average rating of 5.40 on this item. The time to present the solution was quite limited, and the groups that were fairer in time possibly focused on those parts of the solution with a higher weighting (leaving the alternatives for the end without being able to solve them with good quality). Nevertheless, knowing how to divide the work effort to obtain the maximum profit if there are limitations (like time) is also important.

**Figure 15:**
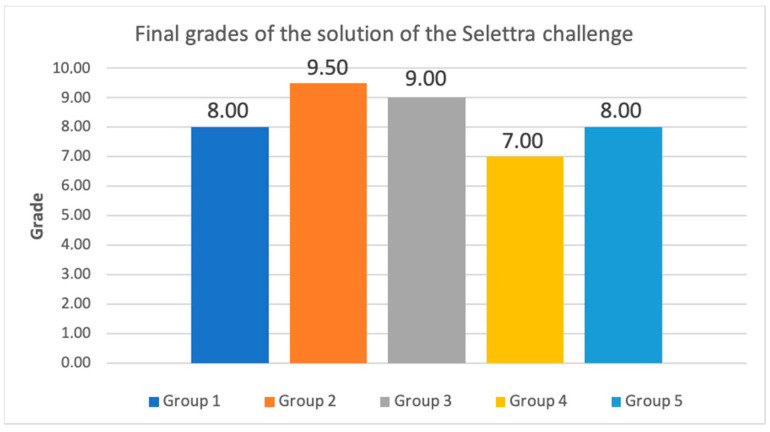
Final grades of the solution of the Selettra challenge.

The final grades of the project are shown in Figure 15. These grades represent a solid acquisition of objectives. Four groups obtained a grade of 8 or higher. Only one group was evaluated below 8. Probably, this group was negatively affected by not having any student from the Smart Cities postgraduate program: a student profile that provided an interesting point of view regarding the viability of the solution and innovation to the rest of the groups.

### 6.2. Student Feedback Analysis

Feedback is an essential part of the learning process and helps to improve the learning experience, by considering the opinion of the students about the subjects they have taken. This feedback allows professors to improve their subjects year by year, and it is a fundamental part of the quality system of our institution.

Students from undergraduate program subjects answer a feedback survey for each subject they take. This feedback includes several questions regarding their satisfaction with the subject, the innovation and practical application of its content, and about the dynamics of the sessions. The following figures show a comparison of the last four-year feedbacks regarding these questions. The rating values range from 1 to 5, 1 being not at all satisfied, 2 being slightly satisfied, 3 being satisfied, 4 being very satisfied, and 5 being completely satisfied.

**Figure 16:**
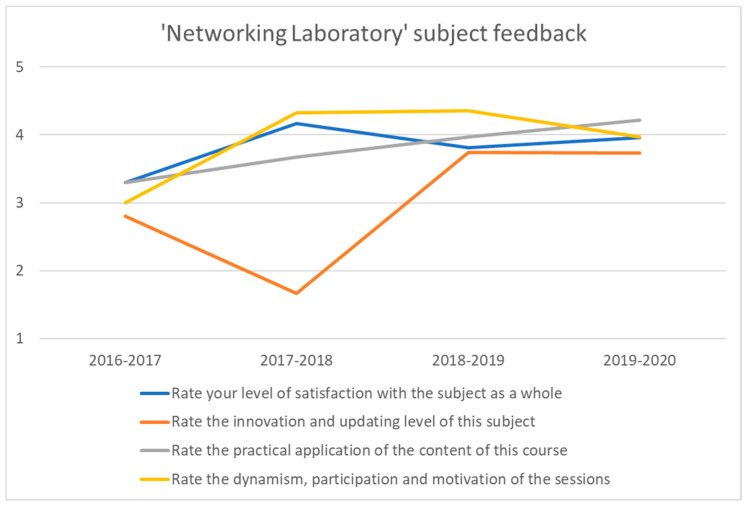
Feedback of the Networking Laboratory subject.

The feedback results of the ‘Networking Laboratory’ subject, shown in Figure 16, are mostly around 4 out of 5. These results show how students are very satisfied with the subject. There was a turning point regarding the innovation of the subject in the course 2017–2018. In this sense, several case studies were created and included in the subject, and the feedback results significantly improved the following year. The practical application of the content is highly rated too, and the mark grows year after year.

**Figure 17:**
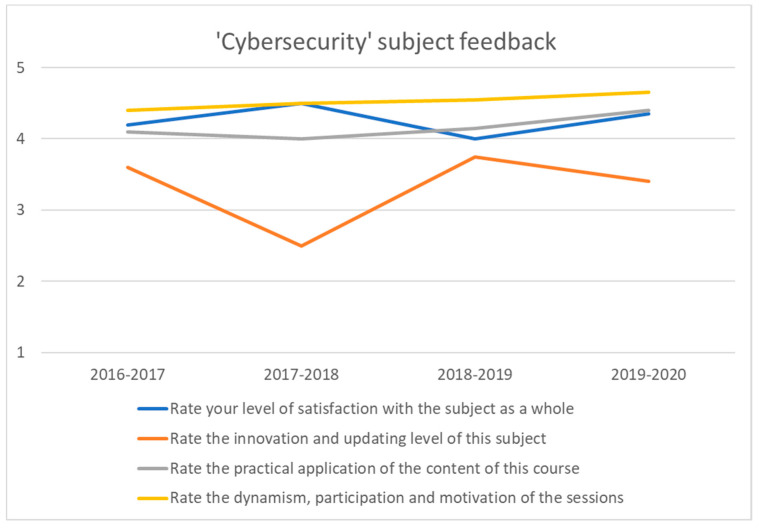
Feedback of the Cybersecurity subject.

The feedback results of the ‘Cybersecurity’ subject shown in Figure 17, are between 4 and 5, apart from the innovation rate. These results show how students are very satisfied with the subject. There was also a turning point regarding the innovation of the subject in the course 2017–2018. In this sense, the subject was updated the following year, and the feedback results significantly improved the following year. The practical application of the content is highly rated, and the mark grows year after year. Additionally, the methodology is very well rated, starting at 4.4 four years ago, and increasing year after year.

Students from advanced stages in a multidisciplinary group were also requested to give feedback about their learning experience. The evaluation forms were mainly addressed to measure the satisfaction of the participants, the fulfillment of their expectations, and the usefulness of the training received in the Data & Security course SPRINT 4.0. 

The presentation of common parts was considered not very interactive, perhaps due to the master class methodology. Regarding the question: “How interactive was the lesson today?”, 75% of the students answered that it had been interactive enough, whereas 25% of the students responded that it had not been very interactive. However, this methodology was confirmed to be very useful in delivering contents. Regarding the question: “How clear were the course objectives?”, 30% of the students answered that they were super clear, 50% answered that they were clear, and the rest responded that they were clear enough.

The audit methodology reached excellent results both in terms of clarity, results achieved, and competencies learned. The Trial explanation was evaluated as an excellent and stimulating case too.

**Figure 18:**
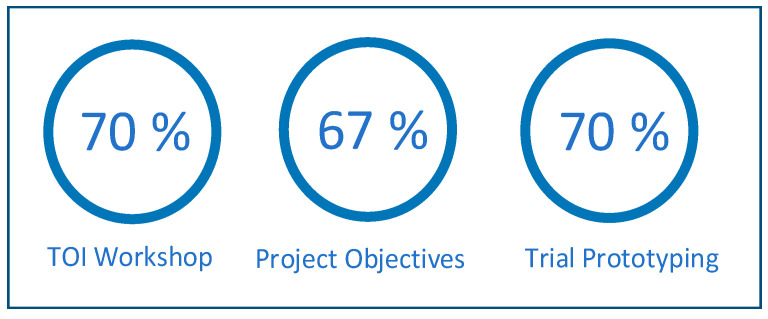
Feedback of the Data & Security course—Sprint 4.0.

The three seminars, Management systems in Industry 4.0; The Big Data lifecycle; and The Cybersecurity challenges in Industry 4.0, reached a good level of satisfaction, a 65% rating. The work project dedicated to the challenge was evaluated very positively: overall, the evaluation was around 70% satisfaction, as Figure 18 shows.

Generally, the activities, contents, and effectiveness reached a high level of satisfaction among participants. Among the success factors, the origin of students from different backgrounds and the possibility to confront real and concrete cases, linking theory and practice, was emphasized. The most critical issue was a theoretical part that was too long, so mixing theory with more hands-on training will be considered for the next courses.

## 7. Conclusions

IoT has become a crucial part of curricula in any engineering curricula related to information and communication technologies, not only in undergraduate programs, but also in master’s courses or any other courses. The cross-cutting nature of IoT needs a different learning approach, capable of fostering the ability to relate different concepts, technologies, and disciplines in an environment in constant evolution. 

An integral pedagogical strategy for learning IoT cybersecurity has been designed and presented in this paper. This strategy takes into consideration the different learning stages of students, depending on their knowledge level of the IoT and their learning maturity. Following the path across the different learning stages will let students gradually improve their knowledge about IoT, and will let them acquire not only technical, but also soft skills. Each stage has been designed to acquire different competencies regarding IoT cybersecurity, by implementing the most suitable learning methodologies. These specific competencies have been carefully designed and included in the learning outcomes of the related programs. The designed mapping between the specific competencies and the IoT reference model has also been presented in this paper, to show the close relationship between these two domains.

This proposed pedagogical strategy needs the definition of the different competencies, but also must go hand in hand with the most appropriate learning methodologies. Therefore, different learning methodologies have been analyzed, and the most suitable learning methodologies have been selected and implemented. Moreover, the learning outcomes expected for each stage have also been defined. Finally, a mapping between the different stages, the IoT cybersecurity competencies, the most suitable learning methodologies, and the expected learning outcomes, has been detailed. 

Different subjects and courses involved in the programs are described in this paper in order to illustrate the effectiveness of this pedagogical strategy. Two different subjects included in the undergraduate degrees, classified in the early stage category, have been detailed: ‘Networking Laboratory’ and ‘Cybersecurity’ subjects. The content, related competencies, the applied learning methodologies, and the expected learning outcomes are specified in-depth for each subject. The student gradebook for the last four years has been analyzed. Student results are very good in both subjects and improve each year. Additionally, the satisfaction feedback provided by the students is included. It can be seen that these subjects are very highly rated, not only regarding the overall satisfaction, but also regarding the innovation, the practical learning outcomes, and the applied methodologies. Moreover, the assessment from other stakeholders, like the companies that are possible future employers and have interviewed the students, has been detailed. Companies marked students with very high grades and revealed an outstanding satisfaction, both with the interviews and the competencies acquired by the students.

The acquisition of competencies in the medium stage is also detailed with the description of the undergraduate program’s final thesis related to IoT cybersecurity and the student collaboration in the Research Group of Internet Technologies and Storage, due to their integration in the different funded research projects of this research group. Finally, an example of an advanced stage experience is presented in the framework of the European Project SPRINT 4.0. The competencies and learning outcomes acquired in this advance stage are detailed. The satisfaction feedback shows very good results in terms of learning outcomes and methodologies. Additionally, the evaluation of the student results of the Selettra challenge shows a very good performance.

The detailed learning experiences show that the presented integral pedagogical strategy for learning IoT cybersecurity adapts to the IoT reference model and integrates the most suitable competencies, methodologies, and learning outcomes.

## Figures and Tables

**Table 1:** Mapping between competencies and IoT Reference Model.

IoT Reference Model Layer	Competency
1—Physical Devices & Controllers	N/A
2—Connectivity	SC.01-1, SC.02-1, SC.02-2, SC.03-1
3—Edge Computing	SC.01-1, SC.03-1, SC.05-1
4—Data Accumulation	SC.01-1, SC.05-1
5—Data Abstraction	N/A
6—Application	SC.04-1
7—Collaboration & Processes	N/A

**Table 2:** Mapping between stages, competencies, learning methodologies and learning outcomes.

Stage	Competencies	Learning Methodologies	Learning Outcomes
Early stages	SC.01-1, SC.02-1 SC.02-2, SC.03-1 GC.02, GC.04, GC.06	LM.01 LM.02 LM.03	LO.01, LO.02 LO.03, LO.04 LO.05, LO.06
Medium stages	SC.01-1, SC.03-1 SC.04-1, SC.05-1 SC.06-1, SC.07 SC.08-1, SC.09-1 SC.10, GC.01, GC.04 GC.05, GC.06, GC.07	LM.02 LM.04	LO.07, LO.08, LO.09 LO.10, LO.11
Advanced stages	SC.08-1, SC.09-1 GC.02, GC.03, GC.04 GC.05, GC.07	LM.02 LM.05	LO.08, LO.09 LO.11

**Table 3:** ‘Networking Laboratory’ Syllabus.

Semester	Title	Description
1st Semester	Introduction to VMWare and Linux	VMWare-based virtualization and basic Linux configuration.
	Routing Exercises	Shows how to perform routing
	Networking & Troubleshooting	Introduction to network concepts and use of sniffers for network troubleshooting in an IoT environment.
	Wire Analysis and Production	Fabrication, testing and reporting of UTP/STP wires with a Fluke Cable Analyzer.
	Cisco ASA Firewalling	Explores the basic configuration and filtering options of a Cisco ASA Firewall used in IoT network architectures.
	Check Point Firewalling	Explores the basic configuration and filtering options of a Check Point Firewall used in IoT network architectures.
	Load Balancing in IoT	Shows how to configure load balancing for IoT services using F5 BIG-IP devices.
	Inter-VLAN routing in IoT	Inter-VLAN routing configuration for IoT network architectures with Cisco Catalyst Devices.
	IPv6 in IoT	Configuration of IPv6 end-devices and networks for the IoT environment.
	WAN Technologies	Configuration of WAN routers to interconnect IoT private networks.
2nd Semester	Introduction to Amazon Web Services	Cloud and network configuration and device virtualization with AWS.
	Wireless Networks for IoT	Implementation of different wireless networks for the IoT environment.
	Data Centers for IoT	Design and configuration of Data Center networks for IoT data processing with Cisco Nexus 7000 devices.
	Advanced Networking with Linux	Configuration of advanced networking and security features in Linux systems such as IP Tables.
	Palo Alto Advanced Firewalling	Explores the advanced configuration and monitoring of a Palo Alto Firewall for IoT network architectures, including High Availability, Virtual Routing and Forwarding and Threat Detection.
	Fortinet Advanced Firewalling	Explores the advanced configuration and monitoring of a Fortinet Firewall for IoT network architectures, including User-ID and Virtual Private Networks.
	Check Point Advanced Firewalling	Explores the advanced configuration and monitoring of a Check Point Firewall for IoT network architectures, including Application Blocking, URL Filtering and SSL Inspection.
	Advanced Routing Exercises	Shows how to perform advanced routing in an IoT environment.
	MPLS for IoT	Configuration of MPLS in Cisco Devices for the interconnection of remote private IoT network architectures.

**Table 4:** Description of Scenarios and objectives.

Scenario		Objectives
1	The Attack of the ICMPs	Explore techniques and scenarios based on ICMP attacks with different types of devices (IoT and non-IoT).
2	The Finger of Printing	Explore scenarios based on IP and ICMP fingerprinting techniques with different types of devices (IoT and non-IoT).
3	The Great Escape	Analyzes techniques to avoid firewalls and IDSs/IPSs.
4	The Honey Potter	Explores the world of Honeypots introducing a new scenario into the testbed.
5	The ICE Kingdom	Learn via a guided process how to solve the challenges proposed in virtual machines of DE-ICE project [29] added to testbed.
6	In the Name of Domain	Explore techniques and vulnerabilities based on DNS attacks.
7	The War of Malware	Analyze malware and botnets and implement one in the testbed.
8	The War of Trojans	Analyze the world of Trojans, how to detect them and how to apply effective countermeasures.

**Table 5:** Distribution of competencies and learning outcomes for early stages.

Subject	Competencies	Learning Outcomes
Networking Laboratory	SC.01-1, SC.02-1 SC.03-1, GC.02 GC.04 GC.06	LO.01, LO.02 LO.03, LO.04, LO.05
Cybersecurity	SC.02-2, GC.02 GC.04, GC.06	LO.06

**Table 6:** Distribution of competencies and learning outcomes for medium stages.

Activity	Competencies	Learning Outcomes
Final Thesis	SC.01-1, SC.03-1 SC.04-1, SC.05-1 SC.06-1, GC.01 GC.04, GC.05, GC.06	LO.07, LO.08, LO.09
Collaboration in GRITS research group	SC.07, SC.08-1 SC.09-1, SC.010, GC.04, GC.06, GC.07	LO.07, LO.08, LO.09, LO.10, LO.11

**Table 7:** Rubric for evaluation of the solution of the Selettra challenge provided by students.

Item Evaluated	Description	Weight
Problem description	Verifies that the students have understood the problem and know how to define the challenge proposal in their own words and in an understandable way	20%
Overview/Requirements	Checks that students correctly extract and define the specific requirements that the proposed solution must meet	20%
Technical Solution	Evaluates the technical solution proposed by the students according to its viability	35%
Innovation	Assesses the degree of innovation of the proposed solution	15%
Rejected Solutions/Alternatives	Evaluates the process of searching for different solutions and the ability to choose the best solution according to defined requirements and available resources	10%

## Appendix A

Table A1 shows the laboratory practices performed in the ‘Cybersecurity’ subject carried out in the second semester of the third academic year.

**Table A1:** Objectives of the laboratory practice performed in ‘Cybersecurity’ subject.

Chapter		Objectives
**1**	**Basic Scenarios** (necessary basic concepts to follow the ‘Cybersecurity’ subject adequately)	● Know how to activate and configure Windows IIS and Linux Web and FTP servers. ● Know how to activate, configure and work with Linux SSH servers. ● Analyze 3-way-handshake TCP process, ICMP traffic (also traceroute operation in Windows and Linux) and other types of relevant traffic to understand network operation (with Linux tcpdump tool). ● Identify, create and use TCP/IP sockets (netcat and netstat tools). ● Learn how to avoid password sniffing on Windows and Linux capturing passwords with Wireshark and tcpdump. ● Learn how to avoid ARP spoofing implementing it and capturing the traffic generated.
**2**	**Cryptography**	● Understand and identify symmetric and asymmetric cryptography methods. ● Know SSH protocol versions and their operation and configuration. ● Understand, identify and use hashing algorithms (MD5 and SHA-1). ● Identify how Linux stores passwords and understand methods of password encryption (MD5, Blowfish, DES, SHA-256, SHA-512). ● Know, identify and manage digital certificates. ● Understand and identify HTTPS and SSL operation.
**3**	**Password Cracking**	● Implement password dictionary attacks (with different tools like John the Ripper, Hydra, Medusa, among others) and learn countermeasures to avoid them. ● Implement Password Brute-force attack (with different tools like Brutus) and learn countermeasures to avoid them. Additionally, analyze rainbow tables performing. ● Be aware of ways to obtain people’s passwords and how to avoid it (social engineering, shoulder surfing, phishing, among others).
**4**	**Basic Network Attacks**	● Implement and identify ping of death attack and learn to apply countermeasures. ● Implement and identify smurf attacks and learn to apply countermeasures. ● Implement Web Session Hijacking attack and learn to apply countermeasures. ● Implement SYN flood attack and learn to apply countermeasures.
**5**	**Footprinting**	● Conduct a footprinting (with different tools and methods suggested in Kali Linux and OSINT documentation) and learn how to document it
**6**	**Host Scanning**	● Identify how Nmap works, which information it helps to gather, what traffic it generates and learn to use it. ● Understand the differences between state results in port scanning, identify Nmap port scans and implement a port scan. ● Identify Nmap options for version detection and use Nmap to detect applications and Operating system versions. ● Learn to use Hping as a scanning tool. ● Learn what is and Idle scanning, implement it and propose countermeasures.
**7**	**Fingerprinting**	● Know and implement fingerprinting, and explain IP and ICMP fingerprinting techniques. ● Know, implement and explain techniques for Server Banner Grabbing. ● Know, implement and explain techniques for Client Banner Grabbing. ● Explain what security through obscurity is used for and implement a header obfuscation scenario.
**8**	**Vulnerabilities**	● Know vulnerability databases and find vulnerabilities for a specific application using these databases. ● Know, describe, install and configure Nessus and identify security problems using this tool.
**9**	**Penetrating**	● Understand “Unicode Directory traversal” vulnerability (CVE-2000-0884, MS00-078), use netcat to hack a Windows server with this vulnerability and, determine and apply countermeasures. ● Understand how Metasploit Framework is used to exploit vulnerabilities, use Metasploit Framework to hack into a Windows system and, determine and apply countermeasures. ● Exploit a poorly secured Linux with basic methods, using netcat to open a backdoor and, determine and apply countermeasures. ● Understand SQLi methods, exploit a vulnerable web server using SQLi, use different tools to implement SQLi, implement complementary methods to test a web server and, determine and apply countermeasures.
**10**	**Firewalls**	● Identify different types of firewalls, use different tools for firewall detection and mapping and, detect and map a firewall in a deployed network. ● Understand tunneling methods and implement firewall circumvention using SSH tunneling and reverse SSH tunneling.
**11**	**Hacking WiFi**	● Identify 802.11 management frames and use Aircrack-ng Suite tools to capture and analyze 802.11 management frames. ● Understand how initialization vectors (IV’s) work and use Aircrack-ng Suite tools to crack WEP keys (under revision to add WPA and/or WPA2 cracking techniques).
